# Nano multi-layered HfO_2_/α-Fe_2_O_3_ nanocomposite photoelectrodes for photoelectrochemical water splitting

**DOI:** 10.1016/j.heliyon.2024.e27078

**Published:** 2024-02-24

**Authors:** Mansour Alhabradi, Xiuru Yang, Manal Alruwaili, Asif Ali Tahir

**Affiliations:** a Environment and Sustainability Institute, University of Exeter, Penryn TR10 9FE, United Kingdom; b Department of Physics, Faculty of Science, Majmaah University, Majmaah, 11952, Saudi Arabia; c Department of Physics, Faculty of Science, Jouf University, 2014, Sakaka 42421, Saudi Arabia

**Keywords:** Hematite, RF magnetron sputtering, Physical vapor deposition, HfO_2_ /α - Fe_2_O_3_, Nano-heterostructure, Photoelectrochemical

## Abstract

This study marks a significant stride in enhancing photoelectrochemical (PEC) water splitting applications through the development of a type II nano-heterojunction comprising HfO_2_ and α - Fe_2_O_3_. Fabricated via Physical Vapor Deposition/Radio Frequency (PVD/RF) sputtering, this nano-heterojunction effectively addresses the efficiency limitations inherent in traditional α - Fe_2_O_3_photoanodes. The integration of HfO_2_ leads to a substantial increase in photocurrent density, soaring from 62 μA/cm^2^ for pure α - Fe_2_O_3_ to 1.46 mA cm^−2^ at 1.23 V versus the Reversible Hydrogen Electrode (RHE). This enhancement, a 23-fold increase, is primarily attributed to the improved absorption of photons in the visible range and the facilitation of more efficient charge transfer. The enhanced performance and long-term stability of the HfO_2_/α - Fe_2_O_3_ nano-heterojunction, validated through XRD, XPS, Raman Spectroscopy, EDS, SEM, EIS, and UPS analyses, demonstrate its potential as a promising and cost-effective solution for PEC water splitting applications, leveraging renewable energy sources.

## Highlights

• HfO_2_/α - Fe_2_O_3_ heterostructure photoanode was successfully fabricated via PVD.
• HfO_2_/α - Fe_2_O_3_ photoanode showed a remarkable photocurrent more than 23 times enhancement.
• HfO_2_ has a narrower band gap and an upward conduction band shift.
• Construction of HfO_2_/α - Fe_2_O_3_ photoanode promoted the charge separation and transfer.

## Graphical abstract

**Figure:**
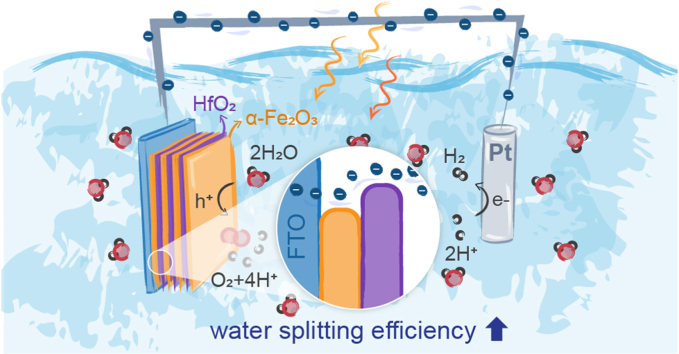

## 1 Introduction

Green hydrogen (H_2_) is a growing potential solution to our global energy and environmental problems [1]. Recent studies indicate that the global population now stands at around 7.9 billion and is projected to reach approximately 9 billion by the year 2050. The consumption of energy is currently ∼25,000 TW-hours per annum. Many reports expect this figure to climb to 40,000 TW-hours per annum [2,3]. Nevertheless, fossil fuels are incapable of satisfying the worldwide need for energy, regardless of the result. Moreover, the emission of greenhouse gases from these fuels does significant harm to the environment.

It is consequently imperative for humanity to begin a search for clean, sustainable, renewable, environmentally friendly, and carbon-free/zero-carbon alternative energy sources with the capacity and efficiency to meet present and future energy needs [4]. Alternative energy sources like solar, wind, geothermal, and hydropower offer a viable foundation for achieving the international goals established by the International Energy Agency (IEA) as outlined in the Sustainable Development Scenario (SDS), aligning with the objectives of the Paris Agreement [5]. Four percent of India's electricity generation in 2020 came from solar. Manufacturing hydrogen (as a fuel) is another option for reducing reliance on traditional sources. Solar energy harvesting is a highly promising and competent alternative for meeting future energy demands [6]. The central topic of this research is a particularly conspicuous instance of this: photoelectrochemical (PEC) water splitting. To create gaseous hydrogen, the ultimate clean fuel, and oxygen via photocatalytic water splitting, solar energy is turned into chemical energy in so-called PEC cells [7,8]. Since Honda and Fujishima made their discovery in the early 1970s of the photo-assisted electrochemical water oxidation of n-type TiO_2_ single-crystal electrodes [9], it has been used to study different semiconducting materials in order to find a suitable material for this application [10]including metal oxides, such as TiO_2_ [11],WO_3_ [12], ZnO [13] and CdO [14]. However, broad band gaps in these metal oxides mean they can only react to UV light, resulting in inefficient use of solar energy [15]. α Fe_2_O_3_(hematite) among these metal oxides has attracted much attention due to its suitable band gap of 2.1 eV [16], chemical stability [17], low cost [18] and non-toxicity [19].The highest theoretical current for hematite is around 12.5 mAcm^−2^ at 1.23 V vs. RHE, making it one of the most effective materials for PEC water splitting [20]. Nevertheless, the photoelectrochemical performance of hematite faces constraints imposed by critical factors, including insufficient electrical conductivity characterized by a hole diffusion length of 2–4 nm and a heightened rate of charge carrier recombination both within the material's bulk and at its surface [21]. As a result, its PEC performance has often been significantly poor [22]. Addressing these limitations involves various strategies, and researchers have found that enhancing the photoelectrochemical performance of hematite anodes is achievable through the formation of nanostructures. These nanostructures include nanorods, nanotubes, nanowires, nanoparticles, and heterostructures formed in conjunction with other semiconductors [7,19,23]. Due to their unique physical and chemical features, heterostructures have received the attention of researchers since they meet the requirements for an effective electrode. Reasonably built heterostructures provide a wide interfacial area that permits quick charge carrier separation, short diffusion length, better light absorption, and improved charge carrier transport [24]. For instance, Luo et al. fabricated a heterostructure of WO_3_/α -Fe_2_O_3_ on an FTO substrate and the result of the PEC performance was shown to be significantly greater than that of WO_3_or α - Fe_2_O_3_ single-component photoanodes due to the efficient separation of electrons and holes [25]. Modifying α -Fe_2_O_3_ nanorods with HfO_2_ overlayer and nanoparticles was controlled using the ALD method [26]. The result demonstrated enhanced PEC performance for water splitting applications. Similarly, a photoanode with a heterostructure of TiO_2_/α - Fe_2_O_3_ was prepared by Jiujun Deng et al. The study reveals that an enhancement of photocurrent by almost 2.1 times because of an improvement in charge migration and separation [27]. Another example, a composite photoanode of BiVO_4_/WO_3_ was designed by Hong et al. and Fujimoto and was shown to be highly active in terms of higher photocurrent generation and (Hydrogen and Oxygen) gas evolution, compared to the BiVO_4_ or WO_3_ alone [28]. Moreover, a study by Panyong Kuang et al. presents a successful implementation of a novel photoanode composed of ternary Fe_2_O_3_/CdS/Co-Pi nanorod arrays. The primary objective of this study and fabrication is to enhance charge separation and improve transfer kinetics both inside the bulk material and at the interface between the electrode and electrolyte [29].

In order to improve the PEC performance of hematite-based photoanodes, several fabrication techniques have been used. These techniques include atmospheric pressure-assisted CVD [30], spray-pyrolysis [31], sol-gel [32], reactive magnetron sputtering [33], aerosol-assisted chemical vapor deposition [23], ferrocene [34], hydrothermal [35,36].

The utilization of radio frequency (RF) stands out as an efficient strategy, known for its exceptional flexibility, uniformity, scalability, and rapid deposition capabilities. This method proves to be particularly suitable for large-scale applications and allows for the swift formation of nanostructures, with the added advantage of easy parameter modification [37]. Wongchoosuk and co-workers studied the fabrication of carbon-doped WO_3_ nanorods by RF magnetron sputtering for NO_2_ gas sensing application. The results show an enhancement in terms of response, response time, selectivity and even working at low temperature [38]. In another example, Qin et al. successfully improved the surface of the Cu_2_O heterojunction photocathode by using the magnetron sputtering for water splitting application [39]. The outcome reveals that the performance of PEC was improved due to the surface densification strategy compared to the others chemical synthesis methods. The photocurrent is increased by roughly 10 times compared to an unaltered device and 1.6 times to that of a typical device utilising electrochemical deposition. Only a few reports are available that illustrated using this method for sputtering metal oxide semiconductors with other semiconductors with heterostructures thin film instead of using traditional chemical methods.

In this report, we demonstrate a novel material that is suitable for energy application by the RF magnetron sputtering method to fabricate a nano-hetero thin film. As has been mentioned above, hematite α - Fe_2_O_3_ is considered to be the most favourable metal oxide for Photoelectrochemical water splitting applications. However, it is limited by some factors and in this work, we have designed a photoanode of HfO_2_/α - Fe_2_O_3_ nano-heterostructures thin film and show its influence on the total PEC performances. Hafnium oxide (HfO_2_) is a promising semiconductor with a wide band gap (Eg **⁓**5.0 eV) dielectric material, high thermodynamic stability and high refractive index [40]. It will play a role in supplying electron tunnelling channels, which will boost the application. Most reports show that the oxygen vacuum conditions required for the targets utilised in the RF magnetron sputtering process significantly raise the production costs associated with this technique. A set of experiments was conducted to identify the most effective conditions for optimizing the photoelectrochemical performance. The impact of deposition parameters, specifically thickness and temperature, on the PEC properties was thoroughly investigated. The samples derived from these experiments underwent a detailed characterization process employing a range of techniques, including X-ray diffraction (XRD), UV–vis absorption spectroscopy, scanning electron microscopy (SEM) with energy-dispersive X-ray spectroscopy (EDX), Raman spectroscopy, and X-ray photoelectron spectroscopy (XPS). The result shows an improvement in the PEC efficiency, and the mechanism was explained in depth, supported by characterization, and verified through experimentation.

## 2 Experimental methdology

### 2.1 Fabrication of HfO_2_/α - Fe_2_O_3_ as nano-heterostructures

**Fig. 1:**
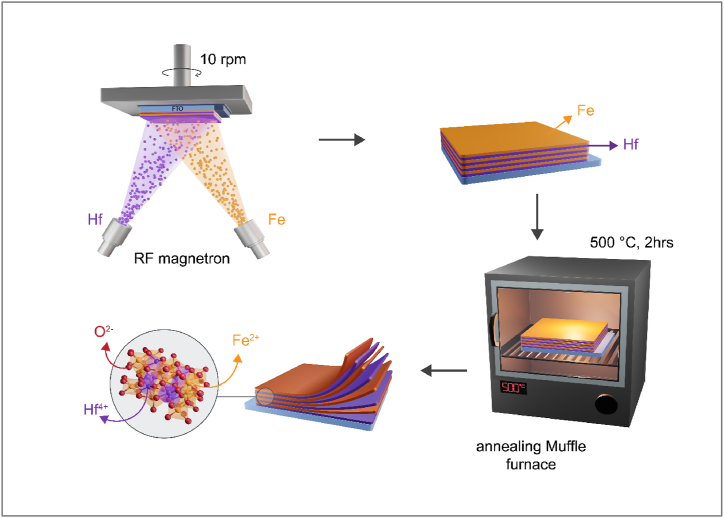
Schematic of RF magnetron sputtering deposition method to fabricate HfO_2_/α - Fe_2_O_3_ film as nano-heterostructures.

Nanocomposite thin films comprised of HfO_2_/α - Fe_2_O_3_ were employed as photoanodes for photoelectrochemical analysis. The fabrication process involved depositing pure iron (Fe) and hafnium (Hf) metal targets on FTO substrate glass with a size of 1 × 2 cm using PVD/RF, as illustrated in Fig. 1. Before to the deposition of nanocomposite films, FTO substrates underwent a thorough cleaning process involving ultrasonication with deionized water and acetone, followed by storage in ethanol.

The space was fixed to be15 cm, between the FTO substrate and the target (ds-t), and the zenithal angle of deposition (α) was maintained at 30° to achieve the desired nano morphology. Deposition is applied under vacuum conditions with a working pressure of 0.3 Pa, using 20 sccm of 99.99% pure Ar gas was supplied into the chamber. The rate of deposition and film thickness were monitored using a quartz crystal microbalance (QCM). Before depositing Fe or Hf onto the substrate, a pre-sputtering process was carried out between 5 and 10 min in argon (Ar) plasma, with the shutter protecting the surface between the target and the substrate. This step aimed to eliminate surface contamination. The sputtering power was set at 65 W and 75 W for Fe and Hf targets, respectively. Throughout the deposition, the substrate rotation speed was fixed at 10 rpm to ensure uniform thickness. The Fe layer was deposited first, followed by the Hf target in a sequence referred to as one cycle. To enhance the performance of PEC, numerous samples were fabricated with varying film thicknesses, ranging from 80 nm (4 cycles) to 240 nm (12 cycles). The fabricated films were heated up from a rang 450 to 600 °C for a duration of 2 h, with a heating rate of 5° per minute in a muffle furnace. This process was carried out to develop a well-constructed heterostructure photoanode. Afterwards, the samples were let to cool naturally to the room temperature before undergoing any analysis or testing. For a fair and comprehensive comparison, For the pure Fe samples, we optimized both the thickness and the annealing temperature. The optimized thickness was set at 100 nm, achieved by adjusting the number of sputtering cycles. The annealing temperature was meticulously chosen to be 600 °C. This temperature was found to be the most effective in achieving the desired crystallinity and phase composition of the α-Fe_2_O_3_, which are critical factors for its PEC performance.

A method for optimizing growth of a thin film with uniform deposition was obtained as reported in our previous paper [14]. Parameters such as pressure and power rating of sputtering were controlled during the processing and optimization was found.

### 2.2 Photoanodes characterization

The crystal structure and phases of the HfO_2_/α - Fe_2_O_3_ nanocomposites were analyzed by a X-ray diffraction with Cu-Kα radiation (λ = 0.154 nm). Confocal Raman spectroscopy was performed using an Alpha300 instrument from WITec GmbH. A visible laser with a wavelength of 532 nm and an output power of 15 mW was employed to excite the sample, while a 50-objective lens collected the backscattered light. The analysis involved averaging ten single spectra. For the examination of surface morphology and elemental composition, scanning electron microscopy (FE-SEM) with TESCAN VEGA3, and energy-dispersive spectroscopy (EDS) with Oxford Instruments were utilised. The film thickness was determined using an FEI Nova 600 Nanolab FIB/SEM system. The optical properties of the coated photoelectrodes were measured over the wavelength range of 200–850 nm using a PerkinElmer LAMBDA 1050 UV/vis/NIR spectrophotometer. X-ray photoelectron spectroscopy (XPS) measurements were conducted with a Thermo Fisher Scientific NEXSA spectrometer. A micro-focused monochromatic Al X-ray source (72 W) was used, covering an area of approximately 400 μm for material analysis. Ultraviolet photoelectron spectroscopy (UPS) was employed to determine the band locations of the samples.

### 2.3 Measurements of PEC performance

The photoelectrochemical performances were carried out using a Newport 66902, 300 W xenon lamp with an AM 1.5 filter in a three-electrode system (Platinum was the counter electrode, Ag/AgCl in 3 M of KCL was the reference electrode, and FTO-coated thin film was the working electrode) on a Metrohm Autolab (PGSTAT302 N) workstation under the 1 sun condition (100mW/cm^2^). As the electrolyte, a 1.0 M NaOH aqueous solution with a pH of 13.6 was utilised. The current-voltage measurements of all the working electrodes were tested within the potential range of (−0.3 V and +0.7) at a scan rate of 0.01 VS^-1^. Throughout the duration of the PEC measurements, each of the electrodes was illuminated from the front, and a working electrode area of 1.0 cm^2^ was maintained. The potential of the working electrode was determined relative to a reversible hydrogen electrode using the Nernst formula (Eq. 1):

(1) $$E_{\text{RHE}}=E_{\text{Ag}/\text{AgCl}}+E_{\text{Ag}/\text{AgCl}}^{0}+0.0591\;V\times \text{pH}\;\left(E_{\text{Ag}/\text{AgCl}}^{0}=0.1976\;V\;\text{vs}\;\text{NHE}\;\text{at}\;25\;{}^{\circ}C\right)$$

In addition, the Mott–Schottky relationship was used in order to calculate the flat band potential (V_fb_) of nanocomposites of HfO_2_/α - Fe_2_O_3_ and pure α - Fe_2_O_3_ films using equation (Eq. 2):

(2) $$\frac{1}{C^{2}}=\frac{2}{e\varepsilon \varepsilon_{0}A^{2}N_{D}}\left(E-E_{fb}-\frac{KT}{e}\right)$$

where C represents the space charge capacitance, and e represents the electron charge (1.6022 × 10^−19^ C). *ε* is the dielectric constant of α - Fe_2_O_3_ (80) [41], ɛ_0_ is the dielectric constant of the semiconductor and permittivity of free space (8.854 × 10^−12^ F/m), A is the area of the thin film, N_d_ is the dopant density, E and E_FB_ are the applied and flat band potential, K_b_ and T are the Boltzmann constant (1.38 × 10^−23^ J/K) and absolute temperature respectively.

## 3 Results and discussion

### 3.1 Characterization of structural and optical properties

**Fig. 2:**
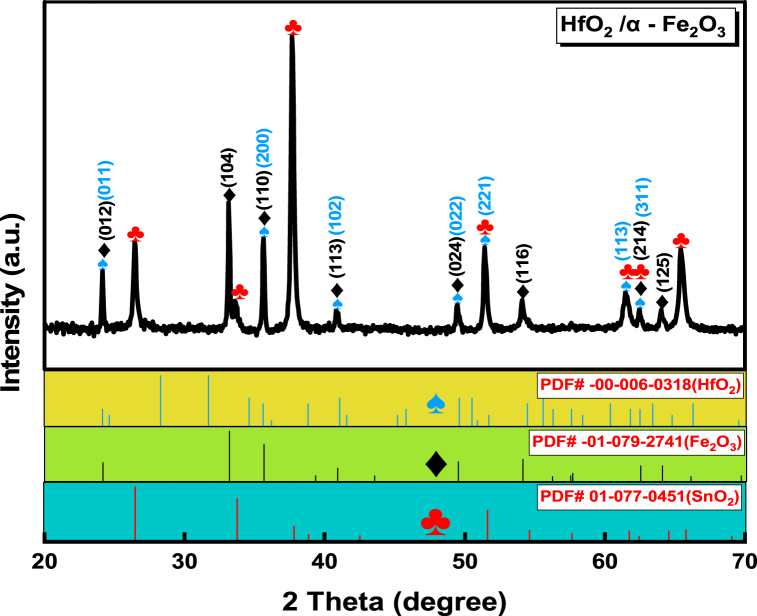
X-ray diffraction patterns of HfO_2_/α - Fe_2_O_3_ as nanocomposites.

Fig. 2 presents the X-ray diffraction patterns of fabricated HfO_2_/α - Fe_2_O_3_ heterostructure thin films on an FTO glass substrate. The crystalline planes that correspond to the peaks for HfO_2_ and α - Fe_2_O_3_ are well indexed in the sample as shown. The diffraction peaks of α - Fe_2_O_3_ could be easily identified as (012), (104), (110), (113), (024), (116), (214), (214) and (125) which corresponds to the hematite phase (PDF# -01-079-2741). Moreover, HfO_2_ corresponding peaks are also detected as (011), (200), (102), (022), (221), (113) and (311) which matches the Monoclinic phase (PDF# -00-006-0318(HfO2)). The strongest peaks of FTO glass are clearly observed from X-ray diffraction patterns which correspond to tetragonal SnO_2_ (PDF# 01-077-0451(FTO)). In the meantime, no other diffraction peaks were found, verifying the exceptional quality of the HfO_2_/α - Fe_2_O_3_ heterostructure films that were fabricated.

**Fig. 3:**
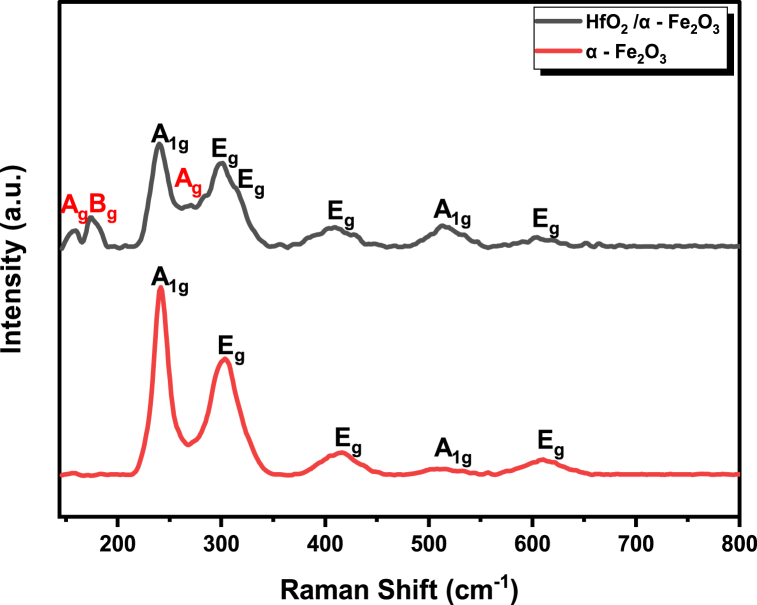
Raman spectra of bare α-Fe_2_O_3_ and HfO_2_/α-Fe_2_O_3_ nano-heterostructure thin films fabricated by PVD.

Raman spectroscopy is an effective tool to examine the structural properties of the fabricated thin films to show different materials phases according to discrete vibrational modes [42]. Fig. 3 shows the Raman spectrum of pristine α - Fe_2_O_3_ and HfO_2_/α - Fe_2_O_3_ that is prepared by PVD. The spectrum of pristine α - Fe_2_O_3_ shows five phonon modes located at 230 and 510 cm^−1^ that correspond to A_1g_ mode, whereas the peaks at 297, 410 and 601 cm^−1^ are assigned to E_g_ Ref. [43]. These findings are in good agreement with the characteristic Raman spectra of α - Fe_2_O_3_, which have a hexagonal shape and exhibit two A_1g_ and three E_g_ phonon modes. These results demonstrate that the α - Fe_2_O_3_ that was fabricated is of high purity, as there are no other peaks observed. The results from the XRD experiment provide even more support for this. The spectrum of heterostructure HfO_2_/α - Fe_2_O_3_ exhibits peaks that are the same or nearly the same wavenumber as those of the obtained α - Fe_2_O_3_ with one extra more peak which is located at 315 cm^−1^ corresponding to E_g_ mode. The Raman spectra of heterostructure thin films also reveal the peaks of monoclinic HfO_2_, which can be seen quite clearly and are located at (A_g_) 153 cm^−1^, (B_g_) 168 cm^−1^ and (A_g_) 268 cm^−1^ modes. This is comparable to the findings that have been published for HfO_2_ in the relevant literature [44,45].

**Fig. 4:**
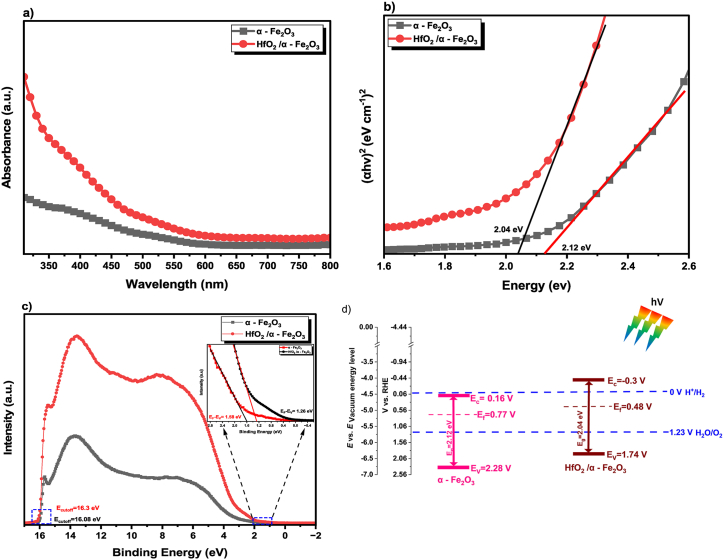
(a) Absorption of HfO_2_/α - Fe_2_O_3_ vs. α - Fe_2_O_3_ (b) Tauc's plot for band gap estimation of bare α - Fe_2_O_3_ (black) and HfO_2_/α - Fe_2_O_3_ (red) nano-heterostructure thin films. (c) Ultraviolet photoelectron spectroscopy (UPS) spectrum of bare α -Fe_2_O_3_ and HfO_2_/α -Fe_2_O_3_ nano-heterostructure thin films. (d) Schematic illustration of the band structures of bare α -Fe_2_O_3_ and HfO_2_/α -Fe_2_O_3_ constructed from UPS.

The optical properties of semiconductors are directly related to their structures and the behaviour of their inner electrons; as a result, these features are acknowledged as important determinants in understanding the photocatalytic activities of semiconductors [46]. UV–Vis spectroscopy was carried out in order to investigate the optical properties of the fabricated photoanode and the band gap via the Tauc plot technique. The HfO_2_/α - Fe_2_O_3_ heterostructures have strong absorption between 300 and 525 nm range, which is indicative of significant electron transitions within the materials. This absorption has a just border difference in the absorption between 525 and 800 nm as shown in Fig. 4 (a). The observed redshift in the absorption edge suggests enhanced light absorption in the visible spectrum, crucial for effective photocatalytic activity. This shift is a clear indication of the impact that doping and the formation of heterostructures have on the band gap. In addition, the values of the band gap energy (E_g_) can be determined from the Tauc equation (Eq. (3)):

(3) $${\left(\alpha hv\right)}^{n}=A\left(hv\;-\;E_{g}\right)$$

where α, hv, n, A and Eg stand for the absorption coefficient, photon energy, n = 0.5 for indirect band gap, and n = 2 for direct band gap materials, the proportionality constant, and optical band gap energy, respectively [47]. Fig. 4 (b) illustrated the band gap values (by drawing tangent a line to the x-axis) of both thin films as bare and heterostructure. For a heterojunction, samples of HfO_2_/α - Fe_2_O_3_ thin films showed a redaction on the band gap value of 2.04 eV, in compared with 2.12 eV of the bare α - Fe_2_O_3_ thin films. Based on this result, it is possible to draw the conclusion that the integration of the HfO_2_ layer, while subtly altering the light absorption characteristics, significantly modifies the electronic properties and the band gap of the base material. These findings underscore the nuanced yet impactful role of HfO_2_ in tuning the optical properties of α - Fe_2_O_3_ in our heterostructure design. Ultraviolet photoelectron spectroscopy (UPS) was used to examine the effect of the HfO_2_ layer on the electronic structure of the HfO_2_/α - Fe_2_O_3_ heterostructures. The band gap positions of the films were calculated by determining the work function (WF) and valence band (VB) with respect to the Fermi level as shown in Fig. 4 (c). The difference between the vacuum energy level and the Fermi level, also known as the work function (WF) and can be calculated by using the equation (Eq. (4)):

(4) $$E_{WF}=h\nu -\left(E_{cutoff}-\left(E_{f}-E_{V}\right)\right)$$

where hν is the energy of the incident He I line of a He discharge lamp used for UPS (21.22 eV) [48], E_cutoff_ is the value of the energy, which was determined by the process of extrapolating the linear portion of the WF spectrum on the X and Y scales and finding the point where they intersected and the energy band difference from fermi level (E_f_) to valence band maximum edge is a linear extrapolation of the low binding energy of the UPS spectra**.** The estimated values of E_cutoff_ for α - Fe_2_O_3_ and HfO_2_/α - Fe_2_O_3_ films are 16.08 eV and 16.3 eV versus evac (vacuum level) respectively as shown in Fig. 4 (c). A linear extrapolation of the UPS spectra of difference Fermi energy level values at 1.58 eV and 1.26 eV for α - Fe_2_O_3_ and HfO_2_/α - Fe_2_O_3_ film is shown in the inset on the right of Fig. 4 (c) respectively. As a result, using the aforementioned equation, the working functions of α - Fe_2_O_3_ and HfO_2_/α - Fe_2_O_3_ films can be estimated at 5.14 eV and 4.92 eV, respectively. Therefore, the Fermi energy level (E_f_) vs the Vacuum level was determined to be −5.14 and −4.92 eV for thin films α - Fe_2_O_3_ and HfO_2_/α - Fe_2_O_3_, respectively. The relevant potentials were determined to be 0.70 and 0.48 V vs. RHE, respectively, based on the reference standard (where −4.44 eV vs. Vacuum energy level = 0 V vs. RHE) [49], which is in good agreement with the estimated values that are obtained from the Mott-Schottky plots. The valence band energies (E_V_)were calculated to be −6.72 eV and −6.18 eV versus evac (vacuum level) or 2.28 eV and 1.74 eV (RHE) for α - Fe_2_O_3_ and HfO_2_/α - Fe_2_O_3_ films respectively. Thus, the conduction band energies (E_C_) can be estimated to be −4.60 eV and −4.14 eV versus evac (vacuum level) or 0.16 eV and −0.3 eV (RHE) for α - Fe_2_O_3_ and HfO_2_/α - Fe_2_O_3_ films respectively by using the following equation (Eq. (5)):

(5) $$E_{c}=E_{v}-E_{g}$$

Considering the findings, the fact that the Fermi energy level (E_f_) of both materials is close to their respective conduction bands suggests that the HfO_2_/α - Fe_2_O_3_ films constitute an n–n heterojunction [50,51]. By combining these results from UPS analysis, the location of the overall band edge relative to water splitting potentials is shown in Fig. 4 (d). Importantly, the spectroscopic results show that the band gap reduction and Fermi level shift towards the conduction band of HfO_2_/α -Fe_2_O_3_, which leads to boosted light absorption and improves bulk and surface charge transport and transfer characteristics [52,53]. Upon exposure to light, photons with energy exceeding the semiconductor's band gap are absorbed, leading to the excitation of electrons into the conduction band and leaving holes in the valence band. In our HfO_2_/α -Fe_2_O_3_ system, the more negative conduction band of HfO_2_ compared to α -Fe_2_O_3_ drives the photogenerated electrons from HfO_2_ to α -Fe_2_O_3_. Conversely, the relatively more negative valence band of HfO_2_ facilitates the movement of photogenerated holes from α -Fe_2_O_3_ to HfO_2_. This directional movement of holes and electrons across the heterojunction significantly reduces their recombination likelihood, thereby enhancing photocurrent generation.

**Fig. 5:**
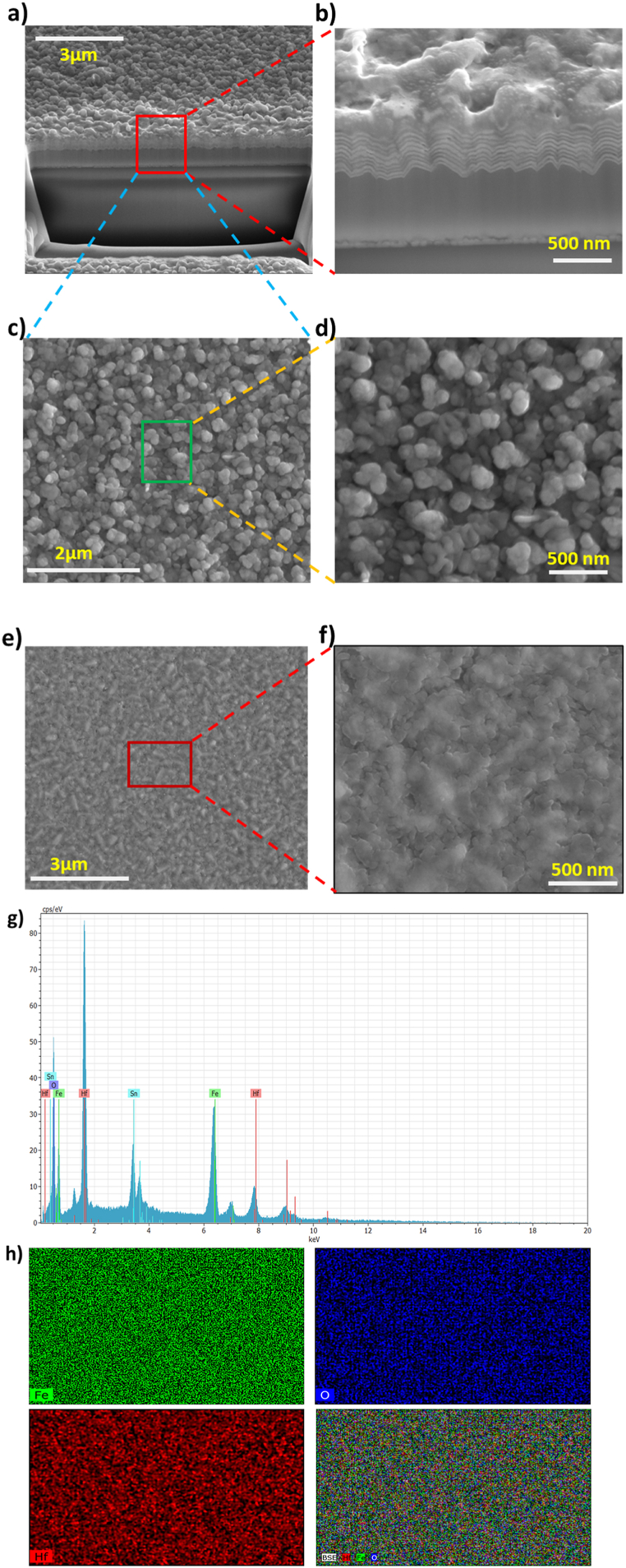
Surface and cross-section FE-SEM images of HfO_2_/α -Fe_2_O_3_ (a, b, s, d) α -Fe_2_O_3_ (g, h). EDS spectra of HfO_2_/α -Fe_2_O_3_ thin films prepared by RF sputtering method (e, f).

The SEM cross-section image of the optimized sample observed for the heterostructure thin film of HfO_2_/α - Fe_2_O_3_ is shown in Fig. 5 (a, b). The result confirmed that the physical vapor deposition (sputtering) method successfully sputters an ultra-thin layer of both metal oxides (Fe, Hf) with better adhesion to FTO glass. The unique layer boundaries of the sputtering could easily be distinguished from the cross-section image as well as showing a good interface between the coating layers. This intergrowth would reduce the negative impacts of electron grain boundary crossing and is believed to be the cause of the longer electron lifetime and decreased recombination within the electrode [54].

Fig. 5 shows top-view SEM images of the surfaces of thin films and their shapes. All SEM pictures show that the film is covered evenly and there are no pinholes. Fig. 5 (c, d) are HfO_2_/α - Fe_2_O_3_ SEM images that show the formation of uniform particle size, from 80 to 200 nm. The large surface area that was fabricated is a sign of the improved electron transport activities of the electrodes as shown in Fig. 5 (d) [12]. There are no cracks in the mesoporous films, but this is different from the thin film of α - Fe_2_O_3_ (single metal), which has a few cracks on the top surface as shown in Fig. 5 (e). The SEM image in Fig. 5 (f) of α - Fe_2_O_3_ thin film exhibits a formation of smoother surfaces overall. In order to validate the stability of the photoanode that was created, the surface of the HfO_2_/α - Fe_2_O_3_ samples was examined using scanning electron microscopy (SEM) images captured for the optimum stability test. Figure S 1 displays images obtained during 7000s of stability testing. Notably, there are no discernible morphological distinctions, except for a minor smoothing of the surface after 7000 s. The presence of Hf and Fe in the modified HfO2/α-Fe2O3 heterostructure can be observed by the EDS mapping in Fig. 5 (g) and the spectra in Fig. 5 (h), and O in the nanocomposite film. However, Sn is detected due to the FTO substrate.

**Fig. 6:**
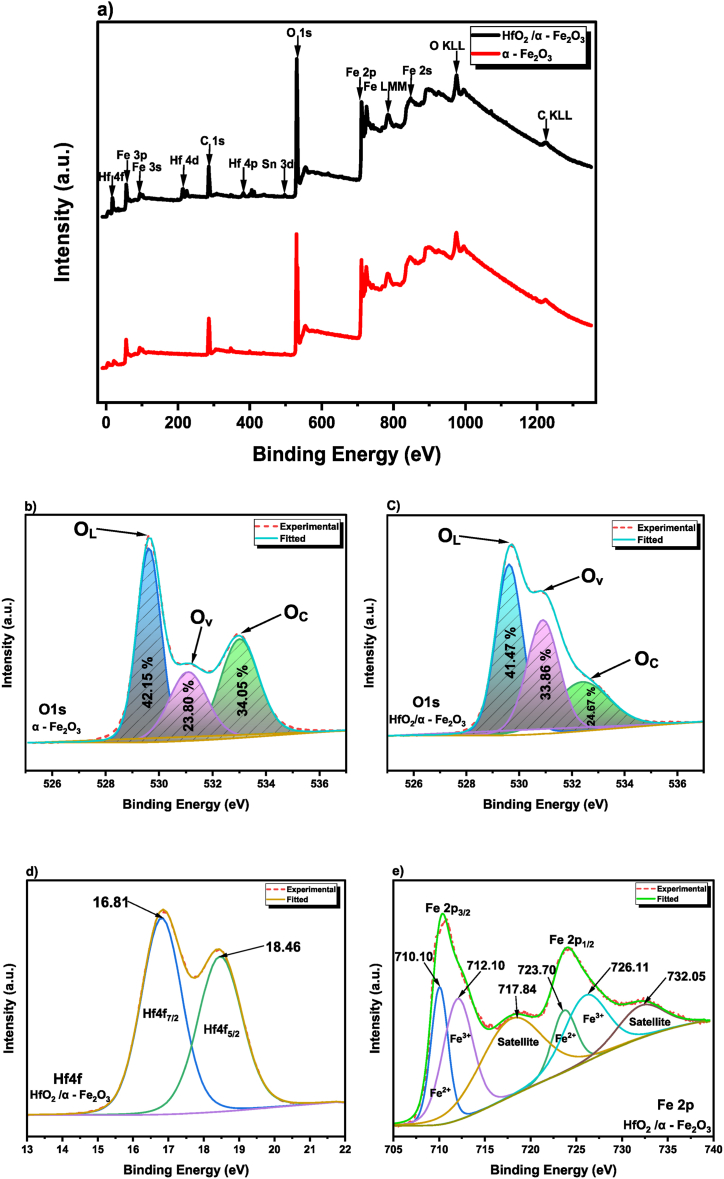
XPS (a) survey spectra of bare α -Fe_2_O_3_ and HfO_2_/α -Fe_2_O_3_; high-resolution spectra of (b) O 1s of α -Fe_2_O_3_. (c) O 1s of HfO_2_/α -Fe_2_O_3_. (d) Hf4f. (e) Fe 2p spectra of HfO_2_/α -Fe_2_O_3_.

To evaluate the surface chemical environment and oxidation state of the optimized sample that were fabricated, the XPS method was utilised. Fig. 6 (a) displays the survey scans of a fabricated α - Fe_2_O_3_ and HfO_2_/α - Fe_2_O_3_ films by the PVD process in the complete range of 0–1400 eV, which demonstrate the existence of Fe, Hf, O, and C elements without any impurity found on the surface of the HfO_2_/α - Fe_2_O_3_ heterostructure thin films. The carbon observed in our XPS spectra, excited using Al kα radiation and calibrated against the C 1s line at 284.5 eV, is due to the calibration process and the C KLL peak results from the Auger process [55]. In addition, the Sn signal occurs from the substrate of the FTO as it has been reported previously [56]. In Fig. 6 (b,c), the O1s spectra of the bare α - Fe_2_O_3_ and HfO_2_/α - Fe_2_O_3_ photoanodes are divided into three types of surface oxygen species [57,58]. These peaks are located at 529.65 eV, 531.09 eV and 532.98 eV which correspond to oxygen atoms lattice (O_L_), oxygen vacancies (O_v_), and adsorbed oxygen species (O_c_), respectively. Hence, O_L_ corresponds to the O^2−^ ion in α - Fe_2_O_3_ or HfO_2_/α - Fe_2_O_3_, and O_v_ may provide additional gas adsorption and reactive sites on the surface of α - Fe_2_O_3_ or HfO_2_/α - Fe_2_O_3_ nanocomposites materials, thus it has a substantial effect on the improvement of PEC performance. On the surface of HfO_2_/α - Fe_2_O_3_ nanocomposites, O_c_ may participate in the redox reaction process [59]. As can be seen in Fig. 6 (b,c), the area % of oxygen atoms lattice O_L_ of bare α - Fe_2_O_3_ and HfO_2_/α - Fe_2_O_3_ photoanodes are 42.15% and 41.47%, respectively. However, the most significant feature is that the area of O_v_ and O_c_ shows the change in the percentages. In compassion, the area of O_v_ increased from 23.80% to 33.86% for α - Fe_2_O_3_ and HfO_2_/α - Fe_2_O_3_ films, respectively. In contrast, O_c_ shows a reduction of the area percentage by 9.38% for HfO_2_/α - Fe_2_O_3_ photoanodes. Importantly, the result verified the presence of oxygen vacancies (O_v_) HfO_2_/α - Fe_2_O_3_ increased which indicates that the HfO_2_ may have led to the formation of more oxygen vacancies on the surface of the HfO_2_/α - Fe_2_O_3_ heterostructures film. Oxygen vacancies would play a significant role in boosting the photocatalytic capabilities via the interface transfer performance [57]. Fig. 6 (d) shows the XPS spectra of Hf4f, which reveals two sharp peaks at 16.81 and 18.46 eV, which is corresponding to Hf4f_7/2_ and Hf4f_5/2_ separately. These values are almost like those reported in the previous literature [60]. In Fig. 6 (e), the high-resolution Fe 2p XPS spectrum of the HfO_2_/α - Fe_2_O_3_ heterostructure is presented, where two prominent peaks are discernible. These peaks split into two doublets, accompanied by satellite peaks, which are characteristic of the spin-orbit coupling in α- Fe_2_O_3_. Specifically, the peaks at 710.10 eV and 712.10 eV correspond to the binding energies of Fe^2+^ 2p_3/2_ and Fe^3+^ 2p_3/2_, while those at 723.70 eV and 726.11 eV are linked to Fe^2+^ 2p_1/2_ and Fe^3+^ 2p_1/2_. The presence of both Fe^2+^ and Fe^3+^ suggests a complex surface chemistry, influenced by factors such as oxygen vacancies – a likely consequence of the high-temperature annealing process – and electron donation from dopant ions, as indicated by Refs. [[61], [62], [63]]. This variance in oxidation states, particularly the presence of Fe^2+^ alongside the dominant Fe^3+^ in α- Fe_2_O_3_, highlights the nuanced changes in the micro-chemical environment of the heterostructure's surface, contributing to its enhanced photocatalytic properties. In figure S 2, a negative shift in the binding energies of α - Fe_2_O_3_ is observed when compared to the HfO_2_/α - Fe_2_O_3_ composite with a range from 0.06 to 0.09 eV relative to α - Fe_2_O_3_. The negative shift in α - Fe_2_O_3_ implies an increase in electron density, indicative of electron transfer from HfO_2_/α - Fe_2_O_3_. These shifts in binding energies are consistent with effective charge separation and electron transfer at the HfO_2_/α - Fe_2_O_3_ heterojunction, enhancing the photocatalytic activity of the composite material [58,64]. Hence, it is possible to validate the successful fabrication of the HfO_2_/α - Fe_2_O_3_ heterostructures film.

### 3.2 Photoelectrochemical activity

**Fig. 7:**
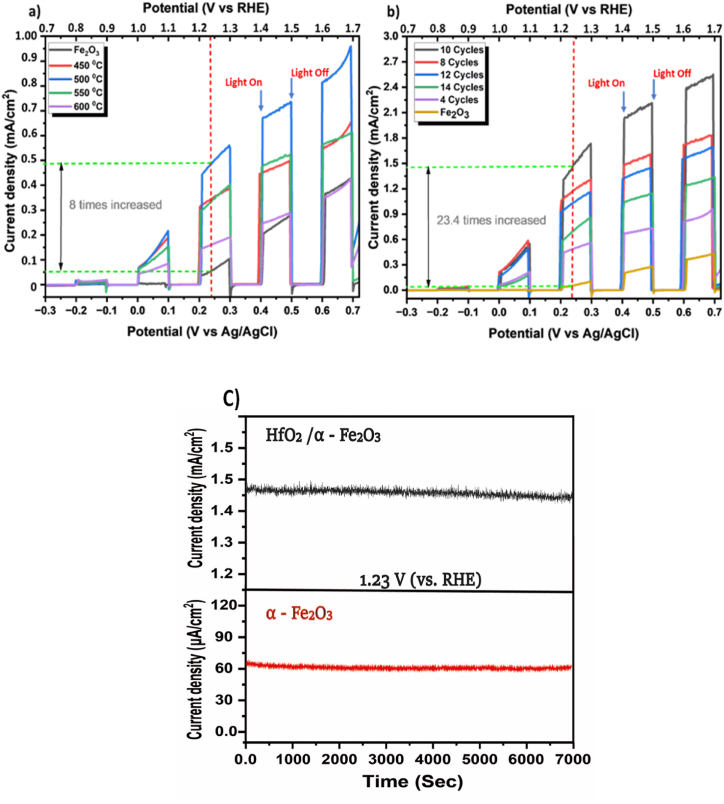
Current-potential characteristics under chopped and light illumination: (a) pure α - Fe_2_O_3_ and HfO_2_/α - Fe_2_O_3_ heterostructure films with different annealing temperatures. (b) various thicknesses of α - Fe_2_O_3_ and HfO_2_/α - Fe_2_O_3_ heterostructure film. (c) Photo-stability of pure α - Fe_2_O_3_ and HfO_2_/α - Fe_2_O_3_ heterostructure film.

To evaluate the Photoelectrochemical water splitting performance under varying annealing temperatures and layer thicknesses, we measured the photocurrent response under AM 1.5 (100 mW cm^2^) simulated solar illumination. Fig. 7 (a) and (b) shows the effect of various annealing temperatures and thicknesses for the heterojunction photoanode. In comparison to the α - Fe_2_O_3_, HfO_2_/α - Fe_2_O_3_ heterostructure films (four cycles) were annealed at 450–600 °C for 2 h as shown in Fig. 7 (a). Evidently, the photocurrent densities of the films clearly increase with increasing temperature, from 150 μA/cm^2^ to nearly 500 μA/cm^2^ at 1.23 V (vs. RHE), whereas pure α - Fe_2_O_3_ produces a photocurrent of 62 μA/cm^2^ at 1.23 V (vs. RHE). The observed over 8 times enhancement in performance, surpassing that of pure α - Fe_2_O_3_ films, can likely be attributed to the enhanced crystallinity of the thin films. This improvement in performance may also be linked to a reduction in defect concentration at various levels, including the bulk, surface, and interface of the fluorine-doped tin oxide (FTO) and HfO_2_/α - Fe_2_O_3_ at the optimized temperature [65,66]. Furthermore, improvement of the device has been achieved by increasing the thickness of the photoanode. The number of layers (cycles) is increased from 4 to 12, as shown in Fig. 7 (b), to study the enhancement of the photocurrent densities of the films. The photocurrent density value tended to steadily grow as the numbers of both layers rose to 10 layers each, and the highest photocurrent density was measured to be 1.46 mA/cm^2^ at 1.23 V (vs. RHE). The value of the photocurrent density, on the other hand, dropped to 1 mA/cm^2^ at 1.23 V (vs. RHE) when the layer numbers were increased more than 10 cycles as shown. It is generally believed that the value of the photocurrent density is closely connected to the heterostructure of the HfO_2_/α - Fe_2_O_3_ layers as a function of their thickness. In terms of photocurrent density, the layer-by-layer HfO_2_ and α - Fe_2_O_3_ structure photoelectrode was the first and most successfully produced. In addition, the increased photocurrent density of HfO_2_/α - Fe_2_O_3_ presents it as an option for future PEC applications, and the obtained results have been compared to those of the other reported materials in the literature as shown in Table S1. As was mentioned before, the existence of the HfO_2_ layer was verified by EDS/SEM measurements. The development of a heterojunction structure was essential in producing an inherent electric field between the junctions. The field of electricity, in return, enabled the acceleration of photoexcited charge carriers. The outcome was a notable reduction in the recombination of electron-hole pairs, ultimately contributing to the observed improvement in photocurrent [51]. The enhancement of photon absorption in the visible region, resulting from the reduction in the optical band gap of HfO_2_/α - Fe_2_O_3_, has led to an increased number of photogenerated electron-hole pairs. This, in turn, has contributed to the realised improvement in photocurrent response. The determination of the kinetics of photoelectrochemical processes relies on the assessment of the Tafel slope. A decrease in the Tafel slope corresponds to an increase in the rate of the electrode reaction. Fig. S3 illustrates that the bare α - Fe_2_O_3_ material demonstrates high Tafel slope values, specifically measuring 750 mV/dec f. In contrast, the nano-heterostructure photoanode has a reduced Tafel slope value of 557 mV/dec, indicating enhanced reaction kinetics in the context of PEC processes. The enhanced photoelectrochemical activity and lower Tafel slope observed in heterostructures are attributed to the rise in charge separation and low recombination rate [67].

Finally, the photoelectrode's stability was another crucial factor in the PEC's performance. The chronoamperometry method was utilised to study the long-term stability of optimized α - Fe_2_O_3_ and HfO_2_/α - Fe_2_O_3_. Both photoanodes were kept illuminated with a fixed applied potential of 0.23 V vs. Ag/AgCl (1.23 V vs. RHE). The results show that the stability of the electrode does not change for up to 7000 s, and the current density is 1.46 mA/cm^2^ for HfO_2_/α - Fe_2_O_3_ and 62 μA/cm^2^ for α - Fe_2_O_3_ as shown in Fig. 7 (C). It is abundantly clear that the α - Fe_2_O_3_ photoelectrode is exceptionally stable in alkaline electrolytes, maintaining approximately 100% of its initial activity.

**Fig. 8:**
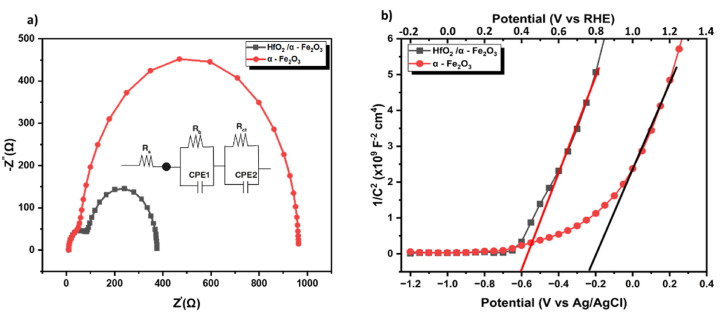
(a) Nyquist plot for EIS measurements obtained for pure α -Fe_2_O_3_ and HfO_2_/α -Fe_2_O_3_ heterostructure films, at 1.23 V vs. RHE under light illumination with the equivalent circuit used for the raw data in inset. (b) Mott-Schottky plots.

Electrochemical impedance spectroscopy (EIS) analyses of the photoanodes yielded further explanations for the enhanced photo-response obtained for HfO_2_/α - Fe_2_O_3_ heterostructure films compared to α - Fe_2_O_3_ films. Electrochemical impedance spectroscopy (EIS) was used to investigate the charge transport mechanisms occurring at both the surface and inside the bulk of the films. Fig. 8 (a) presents the Nyquist plots obtained from the EIS measurements conducted on α - Fe_2_O_3_ and HfO_2_/α - Fe_2_O_3_ photoanodes. The observed data is fitted using a two semicircles equivalent circuit model, as illustrated in the inset of Fig. 8 (a). The related values derived from the fitting circuit are shown in Table S2. The symbol “Rs” in the circuit model symbolises the total resistance in series that includes the resistance of the FTO (Fluorine-doped Tin Oxide) material, the electrolyte, and the conducting wires of the external circuit. The resistance of the charge transport and the capacitance of the space charge in the bulk of the photoanodes are represented by R_b_ and the constant phase element1(CPE1), respectively. On the other hand, the resistance of charge transfer and the capacitance of the interface between the photoanode and electrolyte are described by R_ct_ and CPE2, respectively [68,69]. The values of Rs do not change much after fitting, which shows that the proposed equivalent circuit and fitting method is reliable. In comparison, the charge transport resistance dropped by almost 32% for HfO_2_/α - Fe_2_O_3_ heterostructure compared to pure α - Fe_2_O_3_ film. In contrast, the space charge capacitance considerably increased from 4.77 to 10.06 μF/cm^2^ For α - Fe_2_O_3_ and HfO_2_/α - Fe_2_O_3_ films, respectively, this may facilitate the migration of photogenerated holes to the film surface, where the water-oxygen reaction takes place. Significantly, the charge transfer resistance of the HfO_2_/α - Fe_2_O_3_ films saw a 60% reduction, while the space charge capacitance increased by 51%. Two primary factors have been associated with the substantial decrease in charge transfer resistance. Firstly, the electric field generated at the interface of HfO_2_/α - Fe_2_O_3_, resulting from the formation of a heterojunction structure, significantly enhances charge transport and reduces the recombination of electron-hole pairs, thereby decreasing charge transfer resistance. Secondly, the porous surface of HfO_2_/α - Fe_2_O_3_ provides a larger contact area with more active sites for the oxygen evolution reaction (OER) at the photoanode/electrolyte interface, leading to a significant increase in interfacial charge transfer, resulting in a lower R_ct_ value [59,70]. The EIS result demonstrates conclusively that the fabrication of a HfO_2_/α - Fe_2_O_3_ heterostructure may greatly boost the transfer of photogenerated carriers, resulting in an improved PEC water splitting performance.

The electron density at the devices is related to the rate of charge recombination, as indicated by the electron lifetime value (τ_e_) derived from the Bode plot. A Bode plot depicts the lifetime of an electron at the electrode/electrolyte interface is shown in Fig. S4. The Bode plot, illustrating the log of frequency (Hz) against phase (°), serves as a tool for determining the electron lifetime (τ_e_) in α - Fe_2_O_3_ and HfO_2_/α - Fe_2_O_3_ films, respectively. The estimated value of electron lifetime (τ_e_) was carried by using the following equation (Eq. (6)):

(6) $$\tau_{e}=\frac{1}{2\pi f_{\left(\text{Peak}\right)}}$$

The maximum frequency peak (f_peak_) of bare α - Fe_2_O_3_ and HfO_2_/α - Fe_2_O_3_ films were recorded from the bode plot. The result shows that a shift to a lower value of frequency peak (f_peak_) for α - Fe_2_O_3_ and HfO_2_/α - Fe_2_O_3_ photoanode from 1450 Hz to 150 Hz, respectively. This is a first indication of a longer electron lifetime for the fabrication of a HfO_2_/α - Fe_2_O_3_ heterostructure [71]. The lifetime of electrons of HfO_2_/α - Fe_2_O_3_ films was increased by 8.5-fold in comparison with pure α - Fe_2_O_3_ as shown in Table S2, indicating that the development of the heterojunction structure significantly improves the transfer of charge carriers.

Mott-Schottky analysis was conducted in the dark under conduction for α - Fe_2_O_3_ and HfO_2_/α - Fe_2_O_3_ photoanode, respectively. Fig. 8 (b) illustrates the M − S (Mott-Schottky) curves for both films, aiming to ascertain the donor density (N_D_) and the position of the flat band potentials (E_fb_) in conjunction with their photocurrent response. The outcome reveals a positive slope for both α - Fe_2_O_3_ and HfO_2_/α - Fe_2_O_3_, validating their n-type characteristics and elucidating their photoanodic response [59]. Generally, a higher donor carrier concentration corresponds to increased interface band bending and a heightened driving force for carriers at the semiconductor-electrolyte interface [72]. The flat band potentials (E_fb_) can be estimated from Fig. 8 (b) as −0.23 and −0.6 (V vs Ag/AgCl) or 0.77 and 0.4 (V vs. RHE) for α - Fe_2_O_3_ and HfO_2_/α - Fe_2_O_3_ photoanodes, respectively. Furthermore, the donor density (N_D_) can be calculated using (Eq. (2)) for bare and heterostructure films. The donor density of the HfO_2_/α - Fe_2_O_3_ film is about 1.95 × 10^20^ cm^−3^, which is almost 2 times higher than that of α - Fe_2_O_3_ film (about 1.15 × 10^20^ cm^−3^). This finding demonstrated that the heterojunction successfully increased the PEC performance by increasing conductivity, facilitating the fast transfer of photogenerated carriers, and increasing the degree of semiconductor energy band bending [73].

## 4 Conclusions

In summary, a novel nano-heterostructure of HfO_2_/α - Fe_2_O_3_ film has been designed and fabricated by PVD/RF for photoelectrochemical application. HfO_2_/α - Fe_2_O_3_ nano-heterostructure at 1.23 V vs. RHE obtained a photocurrent density of 1.46 mA/cm^2^, which is almost 23 times greater than that of bare α - Fe_2_O_3_ film as well as showing good stability. Based on XPS, UPS and EIS results confirm the band gap position and the shifting up Fermi level of the HfO_2_/α - Fe_2_O_3_ nano-heterostructure. In addition, oxygen vacancies and a porous structure have the potential to boost the performance of the interface charge transfer. The formation of the nano-heterojunction structure has confirmed the improvement of charge carriers with a reduction of recombination rates. Similarly, the fabricated films show an enhancement of the electron transfer rate at the electrode/electrolyte interface and the effective electron lifetime. The current work has established a straightforward method of fabrication, which, together with its eco-friendliness is highly promising for the development of future water splitting devices.

## Data availability

Data associated with this study have not been deposited into a publicly available repository. Data will be made available on request to the corresponding author.

## CRediT authorship contribution statement

**Mansour Alhabradi:** Writing – original draft, Methodology, Investigation, Data curation. **Xiuru Yang:** Software. **Manal Alruwaili:** Visualization, Methodology. **Asif Ali Tahir:** Writing – review & editing, Visualization, Supervision.

## Declaration of competing interest

The authors declare the following financial interests/personal relationships which may be considered as potential competing interests:Mansour Alhabradi reports financial support was provided by Saudi Arabian Cultural Bureau. Asif Tahir reports financial support was provided by 10.13039/501100000266Engineering and Physical Sciences Research Council. If there are other authors, they declare that they have no known competing financial interests or personal relationships that could have appeared to influence the work reported in this paper.
