# Supplementary material for: Nano multi-layered HfO2/α-Fe2O3 nanocomposite photoelectrodes for photoelectrochemical water splitting
Source: Heliyon. 2024 Feb 24;10(5):e27078. doi: 10.1016/j.heliyon.2024.e27078 (PMC10909753; doi:10.1016/j.heliyon.2024.e27078)
Supplement: Multimedia component 1 [file mmc1.docx]

**Supplementary Material**

**Nanostructured Multi-layered HfO_2_/α-Fe_2_O_3_ Nanocomposite photoelectrodes for Photoelectrochemical Water Splitting**

Mansour Alhabradi^1,2^*, Xiuru Yang^1^, Manal Alruwaili ^1,3^, Asif Ali Tahir^1,^*

^1^Environment and Sustainability Institute, University of Exeter, Penryn TR10 9FE, United Kingdom

^2^Department of Physics, Faculty of Science, Majmaah University, Majmaah, 11952, Saudi Arabia

^3^Department of Physics, Faculty of Science, Jouf University, PO Box 2014, Sakaka 42421, Saudi Arabia

*Corresponding author’s e-mail: Ma943@ exeter.ac.uk, A.tahir@exeter.ac.uk

**Contents of Supplementary**

1. **Comparison of HfO_2_ /α - Fe_2_O_3_ photoanode to other α-Fe_2_O_3_-based photoanodes.**
2. **SEM top view of a HfO2 /α - Fe2O3 film after 7000s of the illumination.**
3. **Fe 2p XPS spectra of the Fe2O3 photoelectrode.**
4. **ECSA normalized Tafel slopes of the Fe2O3 and HfO2 /α - Fe2O3 photoelectrodes. References.**
5. **Parameter values for the optimal EIS curve circuit fitting.**
6. **Fitted Bode plots based on the EIS spectra.**
7. **References.**
8. **Comparison of HfO_2_ /α - Fe_2_O_3_ photoanode to other α-Fe_2_O_3_-based photoanodes.**

**Table S 1: Comparison of HfO2 /α - Fe2O3 photoanode to other α-Fe2O3-based photoanodes.**

| **Photoanode** | **Synthesis method** | **Electrolyte** | **Maximum photocurrent density** | **Reference** |
| --- | --- | --- | --- | --- |
| **Cu_2_S/Fe_2_O_3_** | **Hydrothermal + SILAR** | **1M KOH** | **1.19 mA/cm^2^** **(at 0.92 V vs RHE)** | **[1]** |
| **CuO/Fe_2_O_3_** | **Hydrothermal + an impregnation** | **1 M NaOH** | **0.70 mA/cm^2^** **(at 0.92 V vs RHE)** | **[2]** |
| **Fe_2_O_3_-TiO_2_** | **A facile synthetic** | **1 M NaOH** | **0.2 mA/cm^2^** **(at 0.92 V vs RHE)** | **[3]** |
| **WO_3_/α- Fe_2_O_3_** | **Sputter deposition** | **0.5 M NaOH** | **0.84 mA/cm^2^** **(at 0.92 V vs RHE)** | **[4]** |
| **Mg-Fe_2_O_3_/Fe_2_O_3_** | **Atomic layer deposition (ALD)** | **1M KOH** | **0.5 mA/cm^2^** **(at 1.23 V vs RHE)** | **[5]** |
| **Fe_2_O_3_/CuO** | **dip coating** | **0.5 M Na_2_SO_4_** | **0.53 mA/cm^2^ (at 1.0 V vs. RHE)** | **[6]** |
| **Fe_2_O_3_/BiVO_4_** | **doping method** | **0.5 M Na_2_SO_4_** | **1.3 mA/cm^2^** **(at 1.23 V vs RHE)** | **[7]** |
| **Fe_2_O_3_/Co_3_O_4_** | **Spin-coated /hydrothermal** | **1 M NaOH** | **1.39 mA/cm^2^** **(at 1.23 V vs RHE)** | **[8]** |
| **ZnO/ Fe_2_O_3_** | **chemical solution** | **1 M NaOH** | **1.3 mA/cm^2^** **(at 1.23 V vs RHE)** | **[9]** |
| **Se-doped α- Fe_2_O_3_** | **hydrothermal process** | **0.1 M NaOH** | **0.85 mA/cm^2^** **(at 1.23 V vs RHE)** | **[10]** |
| **HfO_2_ /α - Fe_2_O_3_** | **RF magnetron sputtering process and subsequently thermal oxidation** | **1 M NaOH** | **1.46 mA/cm^2^** **(at 1.23 V vs RHE)** | **Present work** |


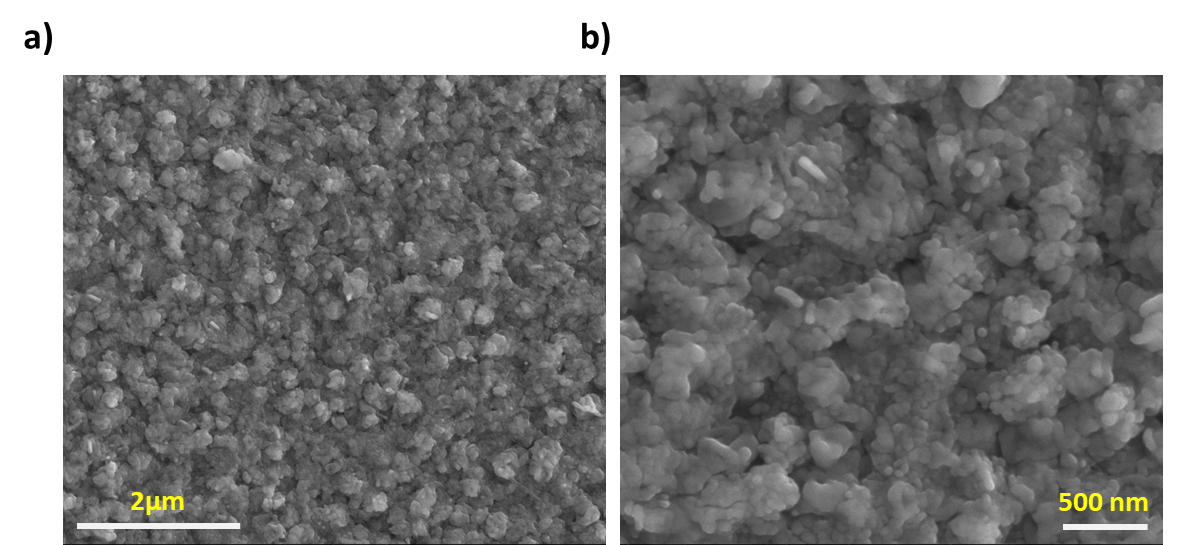


**Figure S 1.** **SEM top view of a HfO_2_ /α - Fe_2_O_3_ film after 7000s of illumination.**

**Figure S 2.** **Fe 2p XPS spectra of the Fe_2_O_3_ photoelectrode.**

**Figure S 3. ECSA normalized Tafel slopes of the Fe_2_O_3_ and HfO_2_ /α - Fe_2_O_3_ photoelectrodes.**

1. **Parameter values for the optimal EIS curve circuit fitting.**

**Table S 2: The determined parameter values for the optimal EIS curve circuit fitting.**

| Photoanode | R_s_  (Ω.cm^2^) | R_bulk_  (Ω.cm^2^) | C_bulk_  (μF/cm^2^) | R_ct_  (Ω.cm^2^) | C_ss_ (μF/cm^2^) | τ_e_  (ms) |
| --- | --- | --- | --- | --- | --- | --- |
| HfO_2_ /α - Fe_2_O_3_ | **9.59** | **78.07** | **10.06** | **288.4** | **56.82** | **1.09** |
| α - Fe_2_O_3_ | **9.36** | **113.80** | **4.77** | **840.4** | **28.01** | **0.106** |

1. **Fitted Bode plots based on the EIS spectra.**

To determine the lifetime of charge carriers, fitted Bode graphs based on EIS spectra are displayed in Figure S 1. The photogenerated charge's lifetime ($\tau\text{e}$) may be computed using the following equation:

$$\tau\text{e}=\frac{1}{2\pi f_{\text{(Peak)}}}$$

where f_peak_ is the maximum frequency peak.

The values that used to calculate the lifetime is shown in the inset of Figure S 1 as zoom in on the maximum peak of both films.

**

**

**Figure S 4. Bode Plots for α - Fe_2_O_3_ and HfO_2_ /α - Fe_2_O_3_ films.**

1. **References**

[1] Y. Zhang, Y. Huang, S.S. Zhu, Y.Y. Liu, X. Zhang, J.J. Wang, A. Braun, Covalent S-O Bonding Enables Enhanced Photoelectrochemical Performance of Cu_2_S/Fe_2_O_3_ Heterojunction for Water Splitting, Small, 17 (2021) 2100320, <https://doi.org/10.1002/smll.202100320>.

[2] J. Ma, Q. Wang, L. Li, X. Zong, H. Sun, R. Tao, X. Fan, Fe_2_O_3_ nanorods/CuO nanoparticles pn heterojunction photoanode: Effective charge separation and enhanced photoelectrochemical properties, Journal of Colloid and Interface Science, 602 (2021) 32-42, <https://doi.org/10.1016/j.jcis.2021.05.140>.

[3] P. Zhang, L. Yu, X.W. Lou, Construction of heterostructured Fe_2_O_3_‐TiO_2_ Microdumbbells for Photoelectrochemical Water Oxidation, Angewandte Chemie, 130 (2018) 15296-15300, <https://doi.org/10.1002/ange.201808104>.

[4] P. Zhao, C.X. Kronawitter, X. Yang, J. Fu, B.E. Koel, WO_3_–α-Fe_2_O_3_ composite photoelectrodes with low onset potential for solar water oxidation, Physical Chemistry Chemical Physics, 16 (2014) 1327-1332, <https://doi.org/10.1039/C3CP53324G>

[5] Y. Lin, Y. Xu, M.T. Mayer, Z.I. Simpson, G. McMahon, S. Zhou, D. Wang, Growth of p-type hematite by atomic layer deposition and its utilization for improved solar water splitting, Journal of the American Chemical Society, 134 (2012) 5508-5511, <https://doi.org/10.1021/ja300319g>.

[6] P.I. Kyesmen, N. Nombona, M. Diale, Heterojunction of nanostructured α-Fe_2_O_3_/CuO for enhancement of photoelectrochemical water splitting, Journal of Alloys and Compounds, 863 (2021) 158724, <https://doi.org/10.1016/j.jallcom.2021.158724>.

[7] L. Wu, M. Wang, T. Han, B. Yang, L. Geng, J. Jin, Fabrication of Fe_2_O_3_/BiVO_4_ heterojunction by doping method to improve the solar water splitting performance of BiVO_4_, Journal of Alloys and Compounds, 949 (2023) 169822, <https://doi.org/10.1016/j.jallcom.2023.169822>.

[8] R. Liang, N. Jiang, Z. Li, Z. Ye, L. Zhu, Amorphous FeO_x_ Overlayer Coupled with Co_3_O_4_ Nanoparticles Passivating Hematite Photoanodes for Water Oxidation, ACS Applied Nano Materials, 6 (2023) 11830-11840, <https://doi.org/10.1021/acsanm.3c01738>.

[9] Y.-K. Hsu, Y.-C. Chen, Y.-G. Lin, Novel ZnO/Fe_2_O_3_ Core–Shell Nanowires for Photoelectrochemical Water Splitting, ACS Applied Materials & Interfaces, 7 (2015) 14157-14162, <https://doi.org/10.1021/acsami.5b03921>.

[10] Z. Ma, Z. Wen, C. Gu, Y. Yin, Doping of Nonmetal Se in Fe_2_O_3_ Nanowire Array-Based Photoanodes for Water Oxidation, ACS Applied Nano Materials, 4 (2021) 13297-13304, <https://doi.org/10.1021/acsanm.1c02807>.
